# Mortality Trends Associated With Acute Myocardial Infarction and Psychoactive Substance Use in Older Adults: A US Nationwide Analysis (1999−2020)

**DOI:** 10.1002/clc.70281

**Published:** 2026-03-25

**Authors:** Noor‐Ul‐Ain Alias Aini, Hasnat Pirzada

**Affiliations:** ^1^ Department of Cardiology Ayub Medical College Abbottabad Pakistan

Dear Editor,

We read with great interest the article, “Mortality Trends Associated With Acute Myocardial Infarction and Psychoactive Substance Use in Older Adults: A US Nationwide Analysis (1999−2020)” published in Clinical Cardiology [1]. Using over 20 years of National data, the authors provide a significant contribution to our knowledge of regional and demographic differences. Given the ongoing opioid crisis, the rise in alcohol abuse, and the increasing incidence of polypharmacy in older adults, their findings are pertinent. However, we believe that a few more restrictions require further consideration to enhance interpretation and future research.

First, the use of ICD‐10 classification lumps together a variety of psychoactive substances under general diagnostic headings, including stimulants, alcohol, opioids, benzodiazepines, and such categorization may conceal risks associated with specific substances. It can also reduce the effectiveness of focused preventive measures [2].

Second, older persons are increasingly using multiple substances, especially alcohol and sedatives or opioids [3]. The risk of AMI may be higher than predicted from single substances alone because of the combined cardiovascular effects of these exposures. However, these interactions cannot be properly accounted for in aggregate mortality data sets.

Third, another unresolved confounding factor is drug−drug interactions. Cardiovascular drugs, such as β‐blockers, anticoagulants, and antiplatelets, are commonly prescribed to older persons. Alcohol or benzodiazepines are examples of psychoactive chemicals that can change pharmacodynamics and cause negative cardiovascular outcomes that are not recorded in death certificate data [4].

Fourth, variations in reporting accuracy, diagnostic thresholds, and coding procedures during the study period may skew the interpretation of temporal mortality trends. Observed patterns may be distorted by differences in the certification of substance use or AMI over time and geographically [5].

Lastly, the nature of this analysis is ecological. The inability to infer individual‐level causality from population‐level death certificate data increases the possibility of ecological fallacy and restricts the ability to account for socioeconomic factors, comorbidities, and access to care [6].

Refined substance category classification, administrative data integration with clinical registries, and the addition of socioeconomic status, treatment quality, and healthcare access metrics are all necessary to overcome these constraints. A more detailed and useful understanding of the relationship between psychoactive substance use and AMI mortality in older persons would result from such methods.

## Data Availability

Data sharing is not applicable to this article as no data sets were generated or analyzed during the current study.

## References

[1] M. H. Shuja , R. Hannat , A. Shahid , et al., “Mortality Trends Associated With Acute Myocardial Infarction and Psychoactive Substance Use in Older Adults: A US Nationwide Analysis (1999–2020),” Clinical Cardiology 48, no. 8 (2025): e70191, 10.1002/clc.70191. 40787966 PMC12337155

[2] R. Alexandrescu , A. Bottle , B. Jarman , and P. Aylin , “Current ICD10 Codes Are Insufficient to Clearly Distinguish Acute Myocardial Infarction Type: A Descriptive Study,” BMC Health Services Research 13, no. 1 (2013): 468, 10.1186/1472-6963-13-468. 24195773 PMC4226256

[3] A. Kuerbis , P. Sacco , D. G. Blazer , and A. A. Moore , “Substance Abuse Among Older Adults,” Clinics in Geriatric Medicine 30, no. 3 (2014): 629–654, 10.1016/j.cger.2014.04.008. 25037298 PMC4146436

[4] S. Liu , S. S. Soedamah‐Muthu , S. C. van Meerten , D. Kromhout , J. M. Geleijnse , and E. J. Giltay , “Use of Benzodiazepine and Z‐Drugs and Mortality in Older Adults After Myocardial Infarction,” International Journal of Geriatric Psychiatry 38, no. 1 (2023): e5861, 10.1002/gps.5861. 36514248 PMC10107716

[5] M. N. Mieno , N. Tanaka , T. Arai , et al., “Accuracy of Death Certificates and Assessment of Factors for Misclassification of Underlying Cause of Death,” Journal of Epidemiology 26, no. 4 (2016): 191–198, 10.2188/jea.JE20150010. 26639750 PMC4808686

[6] H. Morgenstern , “Ecologic Studies in Epidemiology: Concepts, Principles, and Methods,” Annual Review of Public Health 16, no. 1 (1995): 61–81, 10.1146/annurev.pu.16.050195.000425. 7639884

